# Cellular and humoral immunogenicity of the COVID-19 vaccine and COVID-19 disease severity in individuals with immunodeficiency

**DOI:** 10.3389/fimmu.2023.1131604

**Published:** 2023-03-24

**Authors:** C. E. Murray, C. O’Brien, S. Alamin, S. H. Phelan, R. Argue, R. Kiersey, M. Gardiner, A. Naughton, E. Keogh, P. Holmes, S. Naughton, A. Scanlon, A. Sloan, P. McCrea, J. Sui, J. Dunne, N. Conlon

**Affiliations:** ^1^ Department of Immunology, St. James’s Hospital, Dublin, Ireland; ^2^ Wellcome Trust Clinical Research Facility, St. James's Hospital, Dublin, Ireland; ^3^ Department of Biochemistry, St. James’s Hospital, Dublin, Ireland; ^4^ STTAR Bioresource, St. James’s Hospital, Dublin, Ireland; ^5^ School of Medicine, Trinity College Dublin, Dublin, Ireland

**Keywords:** immunodeficiencies affecting cellular and humoral immunity, COVID-19 vaccination, spike protein specific ELISA, IGRA methods, clinical scoring systems

## Abstract

**Background:**

A well-coordinated adaptive immune response is crucial for limiting COVID-19 disease. Some individuals with immunodeficiency are at a high risk of developing severe COVID-19. Therefore, the development of standardized methods for measuring different arms of the vaccine response in the setting of immunodeficiency is of particular interest. In this study, we compared the vaccine response of individuals living with immunodeficiency with healthy controls in terms of interferon gamma (IFN-γ) production and spike protein-specific antibody level post primary COVID-19 vaccination and booster vaccines. Additionally, the disease severity of those individuals who contracted COVID-19 was assessed.

**Methods:**

Whole blood was stimulated overnight from 71 participants and 99 healthy controls. Commercially available PepTivator^®^ peptide pool and trimeric spike protein stimulation were used. ELISA was used to analyze IFN-γ levels. The total SARS-CoV-2 spike protein antibody titre was measured using a Roche Elecsys^®^ S total antibody assay. Patient characteristics, COVID-19 infection status and IDDA 2.1 ‘Kaleidoscope’ scores were recorded. Vaccine responses were scored from zero to three.

**Results:**

99% of healthy controls, 89% of individuals with IEI and 76% with secondary immunodeficiency (SID) had an IFN-γ level above the validated reference range after peptide mix stimulation following primary vaccination. There was an increase in IFN-γ levels in patients with inborn errors of immunity (IEI) following the booster vaccine (p = 0.0156). 100% of healthy controls, 70% of individuals living with IEI and 64% of individuals living with SID had detectable spike protein-specific antibody levels following the primary vaccination. 55% of immunodeficiency patients who had mild COVID-19 and 10% with moderate/severe COVID-19 had detectable antibody and IFN-γ levels post vaccine. The mean pre-infection IDDA 2.1 scores were higher in individuals who developed moderate/severe COVID-19 (25.2 compared to 9.41).

**Conclusions:**

Covid whole-blood IGRA is a highly accurate, straightforward and robust assay and can be easily adapted to measure cellular response to COVID-19. A complete evaluation of the vaccine response may be particularly important for individuals living with immunodeficiency. A clinical immunodeficiency score and a validated vaccine response score may be valuable tools in estimating COVID-19 disease risk and identifying individuals living with immunodeficiency who may benefit from enhanced vaccination schedules.

## Introduction

Since the beginning of the SARS-CoV-2 pandemic, several analytical methods have been developed to measure the host immune response to infection and vaccination. A well-coordinated adaptive immune response is crucial for determining COVID-19 disease severity (1). Consequently, some individuals with primary and secondary immunodeficiency are at a high risk of developing severe COVID-19 (2). Patients with immunodeficiency who might have an impaired humoral and cellular response to vaccination may benefit from enhanced vaccination schedules or be candidates for emerging prophylactic therapeutics. In order to inform such strategies, the development of standardized methods for measuring different arms of the adaptive immune response to vaccines is of particular interest (3, 4).

The most commonly utilized and straightforward method of determining individuals’ vaccine response is measuring SARS-CoV-2 spike protein-specific antibody response following vaccination. It has been demonstrated that the humoral immune response, measured by the SARS-CoV-2 spike protein-specific antibody level, is inversely correlated with disease severity (5–8). However, the durability of antibody response is known to decline over months (9). Neutralizing antibodies are thought to show the greatest potency for host protection (10). Therefore, pseudo-neutralization assays have been developed to measure ACE-2 binding of a pseudo-typed lentivirus and provide a measure of neutralization. However, routine use of such assays in a diagnostic laboratory environment is problematic due to the requirement of tissue culture facilities and lack of standardization of reagents and processes (11).

The cellular response to COVID-19 exposure plays an essential role in sustained immunity and can be measured up to 6- 8 months following SARS-CoV-2 infection, as well as many years after recovery from the related SARS-CoV viruses (12–17). A robust T-cell response has been shown to be associated with less severe COVID-19 disease in healthy and immunocompromised individuals, where an earlier T-cell response may be associated with an improved prognosis (18–20). Additionally, in contrast to antibodies, some evidence suggests that memory T-cells may be cross-protective against different COVID-19 variants and coronaviruses (21, 22). A whole-blood interferon-gamma release assay (IGRA) is a highly sensitive and specific method of measuring cytokine production in response to SARS-CoV-2 infection or vaccination with intact interaction between immune cells simulating *in vivo* conditions (8, 21, 23–25).

Immunological responses to SARS-CoV-2 vaccination have been examined in the setting of immunodeficiency in several ways. To date, numerous studies have investigated the humoral response to SARS-CoV-2 in individuals with immunodeficiency (1). Seven studies have used of ELISPOT, or FluoroSpot assays (2–7, 26), and eight studies have used whole-blood IGRA responses to measure the vaccine response of individuals with immunodeficiency (8–15). Amongst those studies evaluating vaccine response using IGRA, three studies (8, 9, 11) used spike protein, and five studies used peptide (S, S1, S+) mixes including the spike domain (10, 12–15) as antigens. In this study, we sought to investigate the vaccine response in a group of individuals with immunodeficiency in terms of interferon-gamma (IFN-γ) production using a whole-blood assay to spike protein and peptide (S, S1, S+) mix stimulation, in addition to spike protein-specific antibody levels post primary COVID-19 vaccination and booster vaccines. Subsequently, these results were compared with the clinical disease severity of those individuals in the cohort who contracted COVID-19.

## Methods

Ethics approval was granted from the St. James’s Hospital Research Ethics Committee. Healthy controls over 18 years old were enrolled *via* local outreach. Healthy controls were included with no significant co-morbidities, no history of PCR positive COVID-19 disease or COVID-19 like disease. Samples were taken from healthy controls pre-vaccine, 20 – 40 days post completion of primary vaccination course and 28 – 40 days post booster vaccine.

Real-world data were collected from patients recruited from the Immunology outpatient department or day ward from the 1^st^ of February 2021 until the 1^st^ of June 2022. Vaccination of patients was carried out in line with national guidelines. Samples were collected 20 – 40 days following completion of primary vaccination and booster vaccination. Patient characteristics, COVID-19 infection status and Immune Deficiency and Dysregulation Activity 2.1 (IDDA 2.1) ‘Kaleidoscope’ scores were collected and recorded from participants using the electronic patient record. The IDDA 2.1 score is a validated clinical scoring system to measure severity of organ involvement and clinical features in IEI and comprises of 22 parameters on a 2 – 5 point scale (16). Vaccine responses were objectively scored from 0 – 3 for each individual. One mark was given for detectable anti-spike antibodies, IFN-γ production to peptide mix and IFN-γ production to whole spike protein stimulation, respectively. Where COVID-19 infection occurred, disease severity was graded as mild, moderate or severe as per the WHO grading system (17).

1ml of whole blood samples were collected into Qiagen QuantiFERON^®^ Monitor Blood Collection Tubes and stimulated within eight hours of collection. Samples were collected from participants and healthy controls in two separate assays. Commercially available PepTivator^®^ Peptide Pools were used, consisting of spike (pool S, S+ and S1) (0.25 μg/mL) and membrane and nucleocapsid pool (M+N) (0.25 μg/mL), whereby the spike pool was used to measure immune responses to the spike protein of the virus and thus the vaccine response and the nucleocapsid pool was used to measure the immune response to a natural COVID-19 infection. A spike protein trimer (amino acids 14-1213) was also used. Additionally, a Miltenyl Biotec CytoStim^®^ TCR-MHC stimulating reagent (4 µl/ml) was used as a positive control for cytokine expression. Following stimulation samples were incubated for 24 hours at 37°C. After incubation samples were centrifuged @3000 RCF for 15 minutes. Samples were stored at -80°C.

Qiagen QuantiFERON^®^ Monitor ELISA kit was used to analyze IFN-γ levels. The total antibody titre, measuring IgG, IgA and IgM responses to SARS-CoV-2 spike protein was measured from serum using a Roche Elecsys^®^ S total antibody assay. Supernatants were stored for further analysis.

### Statistical analysis

Graphpad Prism was used to for statistical analysis and illustration. The significance level was set at p < 0.05. The Mann-Whitney U test and Wilcoxon test were used to calculate differences in IFN-γ levels between groups. An unpaired *t*-test was used to calculate differences in pre-infection IDDA 2.1 scores between groups.

## Results

### Demographics

In total, 71 patients were included in this study. 44% of individuals with immunodeficiency were female, and the mean age was 47. 48% of healthy controls were female, and the mean age of the healthy controls was 45 years. 65% of the patient cohort was diagnosed with inborn errors of immunity (IEI), and 35% were diagnosed with secondary immunodeficiency (SID). The most common diagnosis of inborn error of immunity (IEI) was common variable immunodeficiency (CVID) (54% of all IEI individuals) followed by X-linked agammaglobulinaemia (XLA) (21%). The remaining 22% of participants with IEI were diagnosed with a heterogenous group of immunodeficiency disorders (**Table 1**). Eight individuals had a history of immunodeficiency secondary to B-cell depletion with anti-CD20 therapy, four individuals were previously diagnosed with lymphoma and thirteen patients were diagnosed with secondary hypogammaglobulinaemia due to other causes. All patients diagnosed with CVID or XLA were maintained on immunoglobulin replacement therapy. SARS-CoV-2 antibodies were not detected in immunoglobulin replacement therapy (Flebogamma, Kiovig, HyQvia and Cuvitru) products in this centre before September 2021, however, some products tested after September 2021 had variable low levels of neutralizing anti-SARS-CoV-2 antibodies.

**Table 1:** Patient characteristics.

Characteristics	Individuals with immunodeficiency n= 71 (Percentage)	Healthy controls n = 99 (Percentage)
**Sex, female (%)**	31 (44%)	49 (49%)
**Age (y), mean**	47	45
Diagnosis		
**Inborn errors of Immunity**	46 (65%)	
XLA	10	
CVID	25	
Post HSCT SCID	1	
CD 40 ligand deficiency	1	
Good syndrome	1	
APECED	1	
DAVID syndrome	1	
Complement deficiency	2	
IgG subclass deficiency	2	
Selective IgA deficiency	1	
Cartilage hair hypoplasia	1	
**Secondary immunodeficiency**	25 (35%)	
Treatment with rituximab	8	
Hypogammaglobulinaemia secondary to other causes	13	
Lymphoma	4	

20% of the total group had no concomitant co-morbidities, 46% of individuals were diagnosed with 1 – 2 co-morbidities, 26% were diagnosed with 3 – 4 co-morbidities, and 8% were diagnosed with ≥ 5 co-morbidities. The most common co-morbidities in the IEI group were bronchiectasis, followed by asthma/COPD, liver dysfunction and autoimmune cytopenia (**Table 2**). Within the group of individuals diagnosed with SID, the most common co-morbidities were asthma/COPD (37% of individuals with SID), lymphoma (29%), cardiovascular disease (29%) and ANCA-associated vasculitis (25%) (**Table 3**).

**Table 2:** Common co-morbidities of the individuals with inborn errors of immunity.

Common co-morbidities	Individuals with inborn errors of immunity (n = 46)
Bronchiectasis	16
Asthma/COPD	8
Liver dysfunction/cirrhosis	5
Autoimmune cytopenia	5
Inflammatory bowel disease	4
Other	29

**Table 3:** Common co-morbidities of the individuals with secondary immunodeficiency.

Common co-morbidities	Individuals with secondary immunodeficiency (n = 25)
Asthma/COPD	9
Lymphoma	7
Cardiovascular disease	7
ANCA-associated vasculitis	6
Chronic kidney disease	4
Bronchiectasis	4
Other	11

### Post vaccine IFN-γ response and spike protein-specific antibody levels in healthy controls

**Figure 1:**
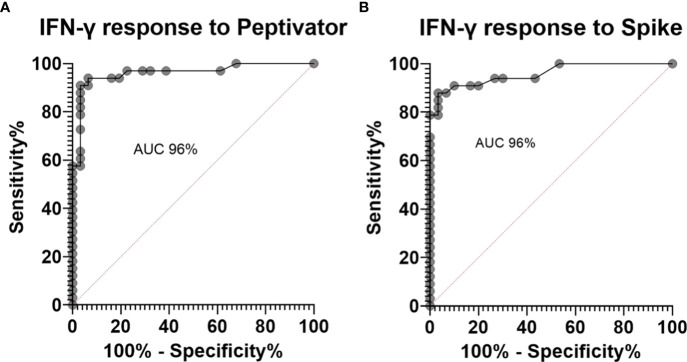
Receiver operator curves for healthy controls pre-vaccination compared to post primary vaccination showing **(A)**. an area under the curve (AUC) of 96% for interferon gamma (IFN-γ) response to peptide (S, S1, S+) mix stimulation and **(B)**. an AUC of 96% for IFN-γ response to spike protein stimulation.

**Figure 2:**
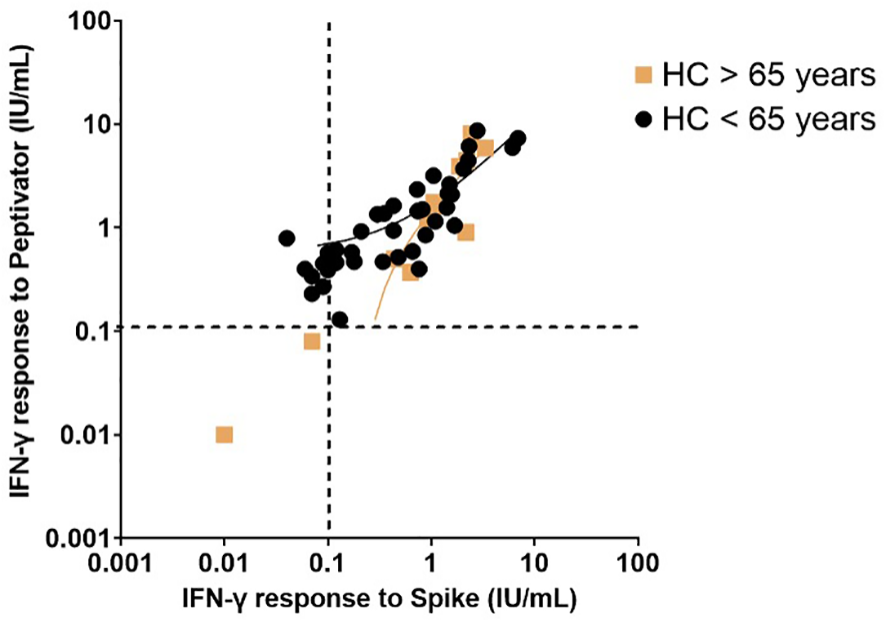
Interferon-gamma response to peptide mix stimulation compared to spike protein stimulation in healthy controls post primary vaccination stratified by age (greater than 65 years compared to less than 65 years). Individual values are plotted on a logarithmic scale (log 10). The dashed line represents the limit of the validated reference range (0.11IU/ml). The solid lines depict the lines of regression.

**Figure 3:**
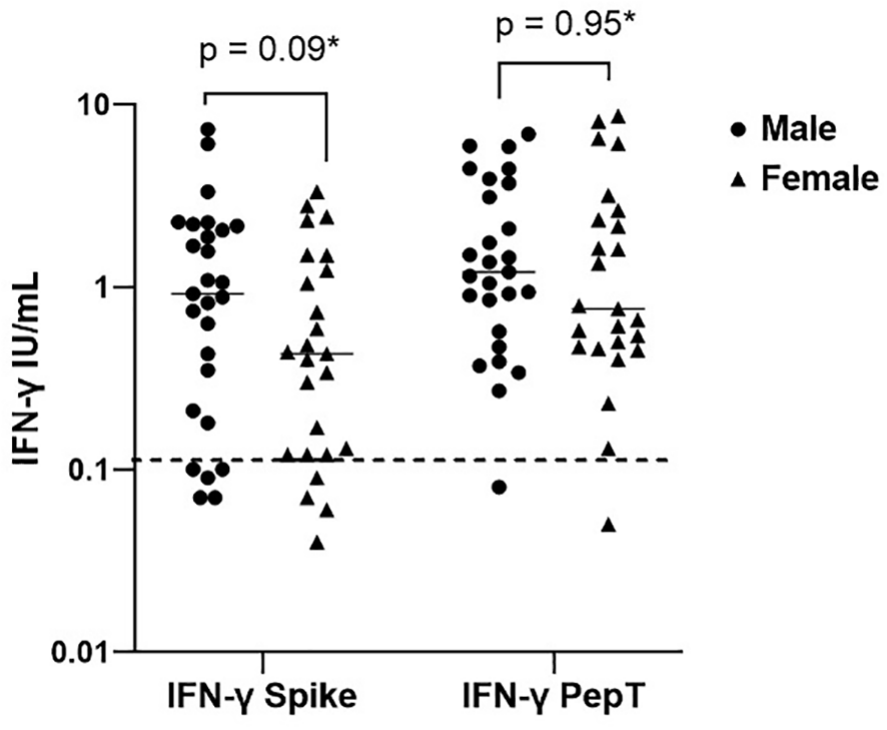
Sex differences in interferon-gamma production post primary vaccination to spike protein and peptide (S, S1, S+) mix stimulation. Individual values are plotted on a logarithmic scale (log 10). The dashed line represents the limit of the validated reference range (0.11IU/ml) and the solid line represents the mean for each group.*Mann-Whitney U test.

**Figure 4:**
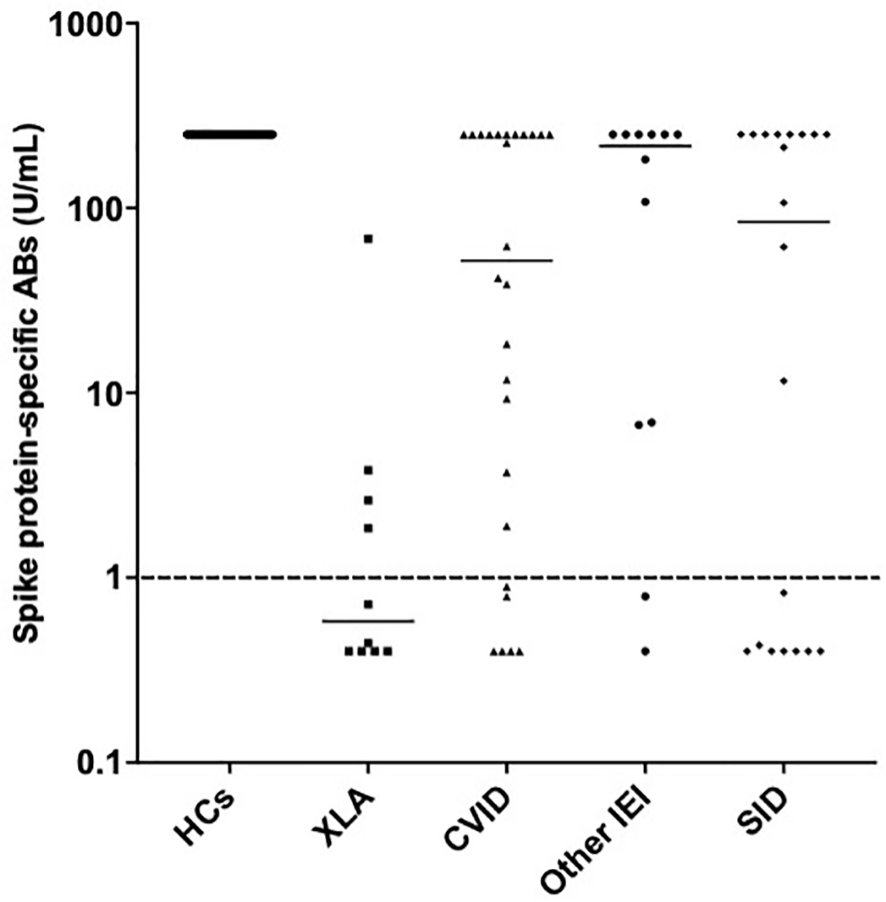
Spike protein-specific antibody responses post primary vaccination in healthy controls, individuals with X-linked agammaglobulinemia, common variable immunodeficiency, other inborn errors of immunity and secondary immunodeficiency. Individual values are plotted on a logarithmic scale (log 10).

34% of the healthy controls received the AZD1222 (AstraZeneca) vaccine and 66% of healthy controls received the BNT162b2 (Pfizer-BioNTech) vaccine. CytoStim™ was demonstrated in all healthy controls and patients with IEI and secondary immunodeficiency, therefore, indicating that T-cell receptor expressing cells were capable of secreting IFN-γ on stimulation. IFN-γ levels to peptide (S, S1, S+) mix were more sensitive than IFN-γ levels to spike protein stimulation (92% compared to 86%, respectively) with comparable specificity (94% and 97%, respectively) in identifying a response to primary vaccination in healthy controls at the validated reference range of 0.11 μg/mL. The area under the curve (AUC) was 96% for both IGRA assays (**Figure 1**). 99% of healthy controls showed adequate IFN-γ secretion above the reference range to peptide (S, S1, S+) mix stimulation, and 92% of healthy controls showed IFN-γ secretion to spike protein stimulation following completion of primary vaccination. There was no overall difference in the level of IFN-γ in the elderly (> 65 years) compared to younger healthy controls (< 65 years) following completion of primary vaccination (p = 0.06). However, two elderly healthy controls had no response to IFN-γ post primary vaccination to peptide (S, S1, S+) mix or spike protein stimulation (**Figure 2**). No significant sex differences were found (p = 0.13 and p = 0.504, respectively) (**Figure 3**). 100% of healthy controls had detectable spike protein-specific antibody levels following completion of the primary vaccination course (**Figure 4**).

### Post vaccination IFN-γ response to membrane and nucleocapsid (M+N) stimulation in healthy controls with and without natural infection

The cut-off 0.1 IU/mL was calculated using the IFN-γ levels after M+N stimulation of 16 post-vaccination (3^rd^ vaccine) healthy controls with no proven infection compared to 9 healthy controls with PCR+ proven infection post 3^rd^ vaccination. At this cut-off the IFN-γ levels after M+N stimulation were 100% sensitive and 94% specific for testing for previous COVID-19 infection. 17% of healthy controls had an IFN-γ level to M+N stimulation above the cut-off at any point. There was no difference in IFN-γ levels to M+N stimulation post vaccination in the healthy controls.

### IFN-γ levels in IEI and SID

89% of individuals with IEI and 76% with SID showed an IFN-γ secretion above 0.11 IU/mL to peptide (S, S1, S+) mix stimulation following completion of primary vaccination. 67% of individuals with IEI and 58% with SID showed adequate IFN-γ secretion to spike protein stimulation following completion of primary vaccination. Of those individuals with IEI with an IFN-γ secretion to peptide (S, S1, S+) mix or spike protein stimulation below the reference range, seven individuals were diagnosed with CVID, and four individuals were diagnosed with heterogenous IEI. Four of the individuals with SID and inadequate IFN-γ production were on anti-CD20 therapy one was diagnosed with lymphoma and four individuals were diagnosed with hypogammaglobulinaemia secondary to other causes.

**Figure 5:**
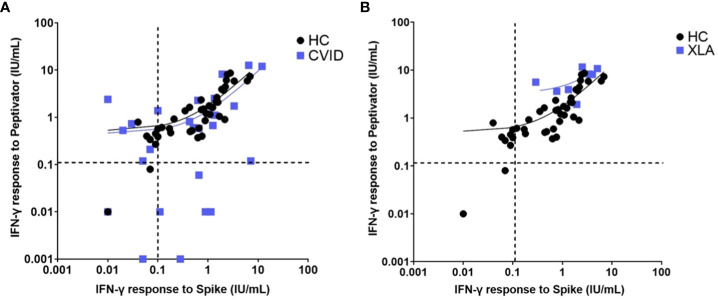
Interferon gamma levels post peptide mix and spike protein stimulation in **(A)**. healthy controls and patients with common variable immunodeficiency and **(B)**. healthy controls and individuals with x-linked agammaglobulinaemia. Individual values are plotted on a logarithmic scale (log 10). The dashed lines represent the lower limit of the validated reference range (0.11IU/ml). The solid lines depict the lines of regression.

**Figure 6:**
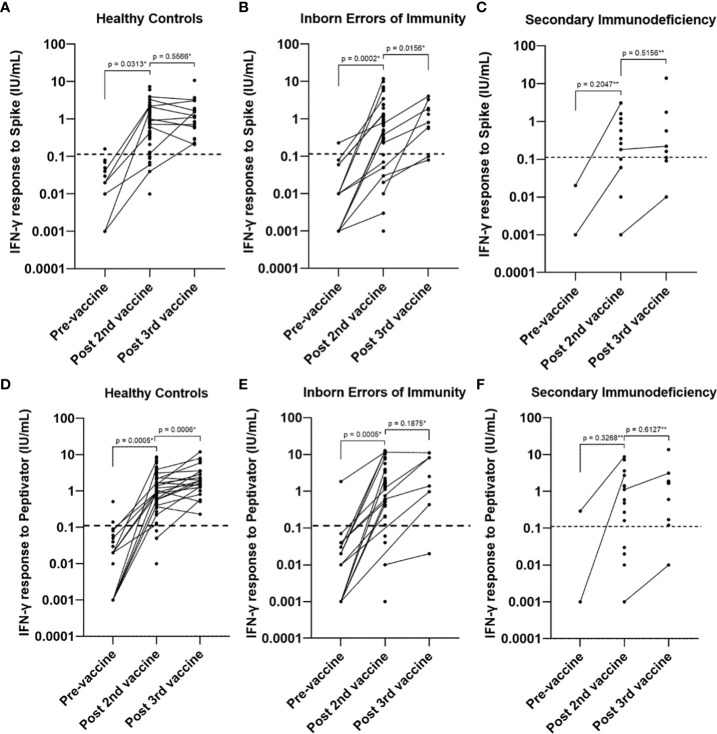
Longitudinal studies of interferon gamma response levels to **(A–C)**. spike protein stimulation and **(D–F)**. peptide (S, S1, S+) mix stimulation pre-vaccine, post primary vaccination and post booster vaccine in healthy controls, individuals with inborn errors of immunity and individuals with secondary immunodeficiency. Individual values are plotted on a logarithmic scale (log 10). The dashed line represents the lower limit of the validated reference range (0.11IU/ml). * Wilcoxon test (paired). ** Mann-Whitney U Test (unpaired).

Patients diagnosed with XLA showed a higher mean IFN-γ level to both peptide (S, S1, S+) mix (6.96 IU/mL in XLA patients compared to 2.05 IU/mL in healthy controls 95% confidence interval [CI] 3.11 – 6.7 p < 0.0001) and spike protein stimulation (2.83 IU/mL in XLA patients compared to 1.18 IU/mL in healthy controls (95% CI 0.42 – 2.87 p = 0.009), after primary COVID-19 vaccination compared to healthy controls (**Figure 5**). There was an increase in IFN-γ levels in patients with IEI following the booster vaccine to spike protein stimulation (p = 0.0156) (**Figure 6**).

### Spike-protein specific antibody level in IEI and SID

70% of individuals with IEI and 64% of individuals with SID had a spike protein-specific antibody response above the reference range (1 U/mL) after completion of primary vaccination. The patient cohort with XLA had the lowest median spike-protein specific antibody level post primary vaccination (0.6 U/mL) compared to CVID (52 U/mL), other IEIs (216 U/mL) and SID (83 U/mL) (**Figure 4**).

### Vaccine response of COVID-19 PCR positive individuals with IEI and SID

Thirty individuals (42% of the total cohort) tested positive for COVID-19 before the data cut-off point. In the total patient cohort, age > 60 was associated with moderate/severe disease (OR 68 95% CI 5.345 – 865.1 p = 0.011). The most common diagnosis amongst COVID-19 positive individuals was CVID (30%), followed by XLA (21%), other heterogeneous IEI disorders (19%) and SID (30%). Ten individuals were diagnosed with moderate-severe COVID-19. Among the individuals with moderate-severe disease, five were diagnosed with IEI, and five were diagnosed with SID.

**Figure 7:**
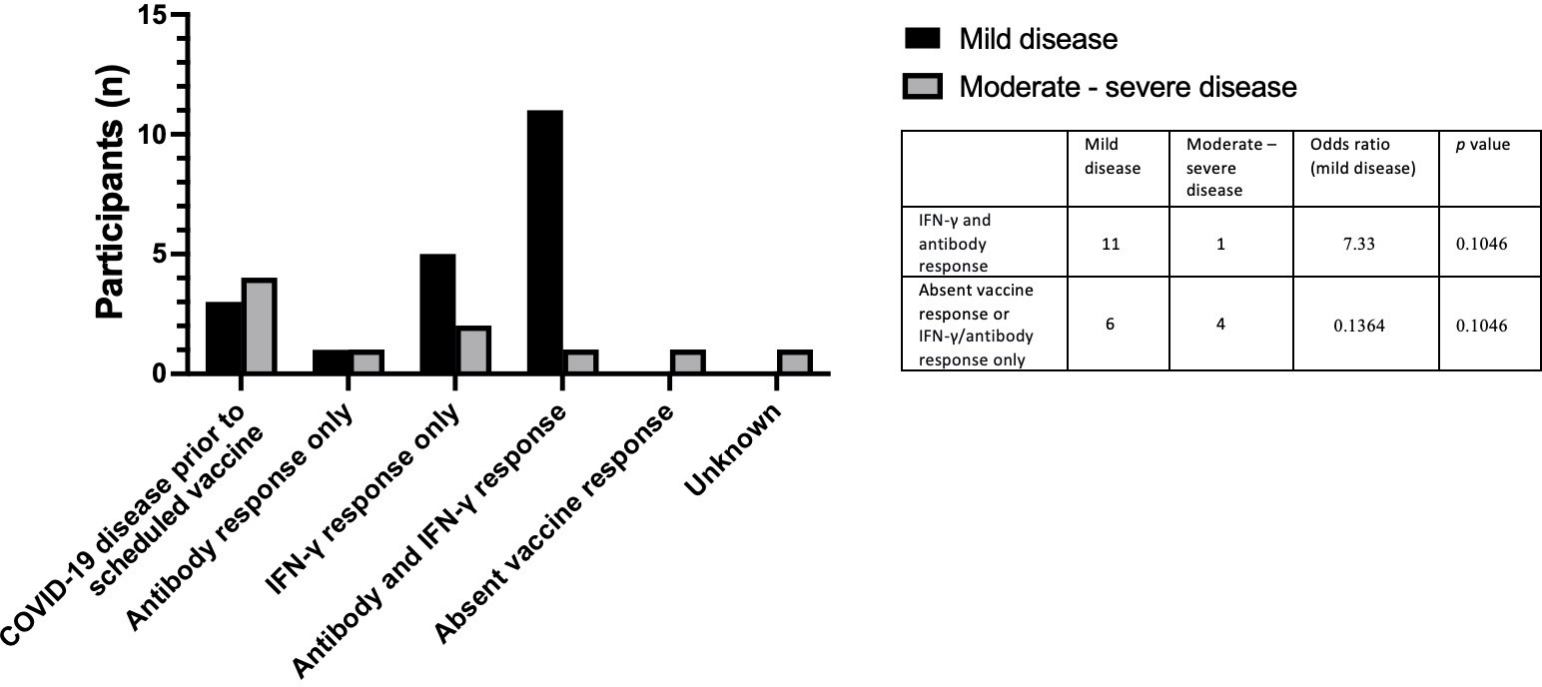
Vaccine responses in individuals testing positive for COVID-19 post vaccination in individuals who developed mild COVID-19 and individuals who developed moderate – severe COVID-19.

Twenty individuals in total were diagnosed with mild COVID-19. The majority of individuals with mild disease (55%) showed a complete vaccine response with a spike protein-specific antibody and an IFN-γ response to peptide or whole spike stimulation. Three individuals with IEI or SID developed mild disease after contracting COVID-19 prior to the scheduled vaccine. Six vaccinated individuals with mild COVID-19 disease had either an IFN-γ or antibody response to vaccination only. Ten individuals with IEI or SID developed moderate/severe COVID-19. Four individuals developed moderate/severe COVID-19 before the scheduled vaccine. One person with IEI or SID diagnosed with moderate/severe COVID-19 disease had a complete vaccine response to all measured modalities (**Table 4**). Individuals with a positive IFN-γ and antibody response were more likely to develop mild disease, however, this was not statistically significant (OR 7.33, p = 0.1046) (**Figure 7**).

**Table 4:** Interferon-gamma responses to spike and peptide mix stimulation, immunoglobulin response post primary vaccination, vaccine response score and COVID-19 disease infection severity of individuals with immunodeficiency.

Diagnosis	Pre-vaccine		Post primary vaccines		Post booster vaccine		Antibody response		Vaccine response score*	Pre-vaccine COVID-19 infection (yes/no)	Disease severity
	IFN-γ Spike	IFN-γ peptide mix	IFN-γ Spike	IFN-γ peptide mix	IFN-γ Spike	IFN-γ peptide mix	Post primary vaccines	Post booster vaccine			
Inborn errors of immunity											
XLA											
Patient 1	3.01	1.82	9.32	6.64	19.37	10.99	68.3	N/A	N/A	Yes	Mild
Patient 2	0.06	0.001	1.32	3.92	N/A	N/A	0.44	N/A	2	No	Mild
Patient 3	0.08	0.02	3.46	8.28	N/A	N/A	1.85	N/A	3	No	Mild
Patient 4	N/A	N/A	5.1	10.79	N/A	N/A	1.44	N/A	3	No	–
Patient 5	N/A	N/A	0.76	1.23	4.04	8.12	0.72	N/A	2	No	Mild
Patient 6	N/A	N/A	0.29	5.63	N/A	N/A	0.4	N/A	2	No	–
Patient 7	N/A	N/A	0.77	7.62	N/A	N/A	0.4	N/A	2	No	–
Patient 8	N/A	N/A	1.95	10.92	N/A	N/A	0.4	N/A	2	No	Severe
Patient 9	N/A	N/A	2.52	11.6	N/A	N/A	3.81	N/A	N/A	Yes	Mild
Patient 10	N/A	N/A	N/A	N/A	N/A	N/A	0.4	N/A	N/A	No	–
CVID											
Patient 11	0.63	2.29	0.51	0.6	N/A	N/A	N/A	N/A	N/A	Yes	Severe
Patient 12	0.001	0.04	0.51	0.6	N/A	N/A	250	N/A	3	No	–
Patient 13	0.001	0.001	0.02	0.53	N/A	N/A	0.4	N/A	1	No	–
Patient 14	0.06	0.07	1.36	1.11	N/A	N/A	250	N/A	3	No	Mild
Patient 15	0.001	0.001	0.47	2.23	N/A	N/A	225	N/A	3	No	–
Patient 16	0.23	0.001	0.81	0.43	N/A	N/A	0.4	N/A	2	No	Mild
Patient 17	0.01	0.02	6.49	12.62	N/A	N/A	9.31	N/A	3	No	Mild
Patient 18	0.01	0.03	11.96	11.96	N/A	N/A	250	N/A	3	No	–
Patient 19	0.01	0.01	N/A	N/A	0.61	0.96	N/A	62.2	3	No	–
Patient 20	0.01	0.01	0.07	0.21	N/A	N/A	250	N/A	2	No	Mild
Patient 21	N/A	N/A	5.73	10.21	N/A	N/A	250	N/A	3	No	–
Patient 22	N/A	N/A	0.01	0.01	N/A	N/A	18.4	N/A	1	No	–
Patient 23	N/A	N/A	0.11	0.42	N/A	N/A	41.9	N/A	3	No	Mild
Patient 24	N/A	N/A	0.03	0.001	N/A	N/A	0.4	N/A	0	No	–
Patient 25	N/A	N/A	1.14	1.83	N/A	N/A	38.8	N/A	3	No	–
Patient 26	N/A	N/A	2.4	3.24	N/A	N/A	250	N/A	3	No	–
Patient 27	N/A	N/A	0.28	0.53	N/A	N/A	0.4	N/A	2	No	–
Patient 28	N/A	N/A	0.89	1.43	N/A	N/A	250	N/A	3	No	–
Patient 29	N/A	N/A	0.38	0.75	1.92	8.21	250	250	N/A	Yes	Mild
Patient 30	N/A	N/A	0.01	0.01	0.01	0.02	0.4	1.9	N/A	Yes	Moderate
Patient 31	N/A	N/A	0.05	0.12	N/A	N/A	0.79	N/A	0	No	–
Patient 32	N/A	N/A	0.02	0.61	0.1	1.38	0.9	3.21	2	No	–
Patient 33	N/A	N/A	0.66	0.58	N/A	N/A	250	N/A	3	No	–
Patient 34	N/A	N/A	N/A	N/A	1.34	2.51	N/A	11.8	3	No	Mild
Patient 35	N/A	N/A	0.001	0.001	N/A	N/A	0.4	N/A	0	No	–
CD40 ligand deficiency											
Patient 36	0.001	0.001	0.03	0.17	0.08	0.43	6.92	N/A	2	No	Mild
Good syndrome											
Patient 37	0.001	0.04	0.32	0.51	N/A	N/A	N/A	N/A	N/A	No	Severe
Cartilage hair hypoplasia											
Patient 38	N/A	N/A	0.03	0.04	N/A	N/A	250	N/A	1	No	–
C2 deficiency											
Patient 39	N/A	N/A	0.74	1.94	N/A	N/A	250	N/A	3	No	–
C7 deficiency											–
Patient 40	N/A	N/A	N/A	N/A	0.56	1.74	N/A	108	3	No	Mild
DAVID syndrome											
Patient 41	N/A	N/A	0.001	0.2	N/A	N/A	6.7	N/A	2	No	–
IgG subclass deficiency											
Patient 42	N/A	N/A	0.24	0.53	N/A	N/A	250	N/A	3	No	Moderate
Patient 43	N/A	N/A	0.3	1.56	N/A	N/A	250	N/A	3	No	Mild
APECED											
Patient 44	N/A	N/A	1	1.68	N/A	N/A	0.4	N/A	2	No	–
Post HSCT SCID											
Patient 45	N/A	N/A	0.05	0.06	N/A	N/A	183	N/A	1	No	Mild
Selective IgA deficiency											
Patient 46	N/A	N/A	2.44	7.51	N/A	N/A	250	N/A	3	No	Mild
Secondary immunodeficiency											
Hypogammaglobulinaemia secondary to lymphoma											
Patient 47	N/A	N/A	0.1	0.19	N/A	N/A	250	N/A	3	No	–
Patient 48	0.02	0.29	3.05	8.74	N/A	N/A	250	N/A	N/A	Yes	Severe
Patient 49	N/A	N/A	N/A	N/A	13.83	13.7	250	250	N/A	Yes	Moderate
Patient 50	N/A	N/A	0.32	0.39	N/A	N/A	250	N/A	3	No	–
Treatment with rituximab											
Patient 51	0.001	0.001	0.06	2.73	N/A	N/A	0.4	N/A	1	No	–
Patient 52	N/A	N/A	0.01	0.59	N/A	N/A	0.83	N/A	1	No	Mild
Patient 53	N/A	N/A	0.19	0.001	N/A	N/A	0.4	N/A	1	No	Mild
Patient 54	N/A	N/A	0.18	1.12	0.22	3.12	0.4	N/A	2	No	Moderate
Patient 55	N/A	N/A	1.6	3.6	N/A	N/A	0.4	N/A	2	No	–
Patient 56	N/A	N/A	0.35	1.43	N/A	N/A	0.43	N/A	3	No	–
Patient 57	N/A	N/A	0.9	1.38	N/A	N/A	0.4	N/A	2	No	–
Hypogammaglobulinaemia secondary to other causes											
Patient 59	0.001	0.001	0.01	0.01	N/A	N/A	107	250	1	No	Moderate
Patient 60	N/A	N/A	0.26	0.16	N/A	N/A	213	N/A	3	No	–
Patient 61	N/A	N/A	0.001	0.001	N/A	N/A	0.4	N/A	0	No	Severe
Patient 62	N/A	N/A	0.06	0.27	N/A	N/A	61.7	N/A	3	No	–
Patient 63	N/A	N/A	N/A	N/A	0.11	0.01	N/A	250	2	No	–
Patient 64	N/A	N/A	N/A	N/A	0.09	0.12	N/A	250	2	No	–
Patient 65	N/A	N/A	N/A	N/A	0.16	0.17	N/A	250	3	No	–
Patient 66	N/A	N/A	N/A	N/A	1.83	1.65	N/A	0.8	2	No	–
Patient 67	N/A	N/A	0.01	0.01	N/A	N/A	0.4	N/A	0	No	–
Patient 68	N/A	N/A	0.18	1.3	N/A	N/A	250	N/A	3	No	Mild
Patient 69	N/A	N/A	N/A	N/A	1.75	1.89	N/A	250	3	No	–
Patient 70	N/A	N/A	0.57	0.48	N/A	N/A	N/A	N/A	N/A	No	–
Patient 71	N/A	N/A	1.18	7.16	N/A	N/A	250	N/A	3	No	–

*Vaccine responses were scored from 0 – 3 for each individual. One mark was given for detectable anti-spike antibodies, IFN-γ production to peptide mix and IFN-γ production to whole spike protein stimulation following primary or booster vaccines, respectively. Unavailable data is annotated as not available (N/A).

### IDDA 2.1 ‘Kaleidoscope’ scores in COVID-19 positive individuals with IEI

**Figure 8:**
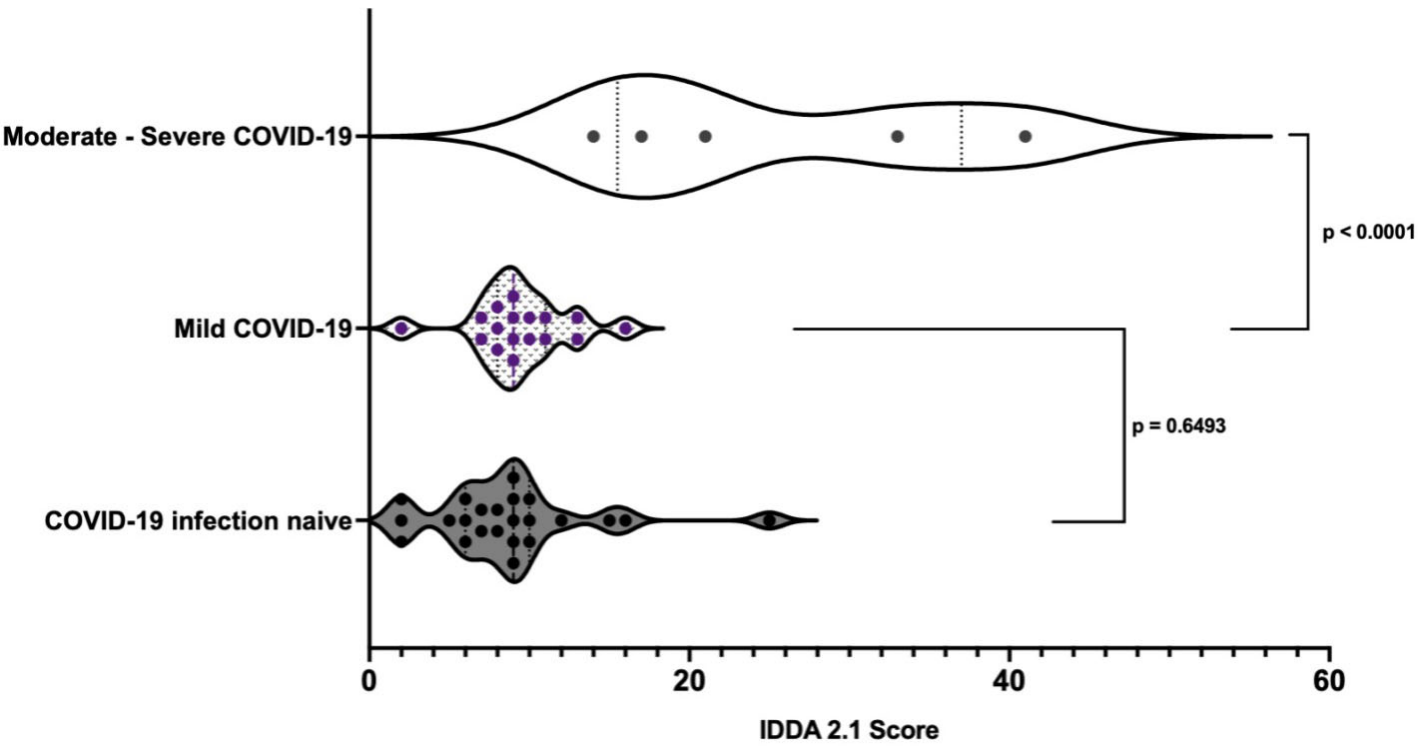
Pre-infection immune dysregulation and disease activity 2.1 scores in those individuals with IEI stratified by COVID-19 disease severity.*Unpaired *t*-test.

No difference was found in the IDDA 2.1 scores between infection naïve individuals and those who developed mild COVID-19 symptoms (p = 0.6493). IDDA 2.1 scores of those who developed moderate/severe COVID-19 were higher on average than individuals who developed mild COVID-19 (25.2 95% CI 11.03 – 39.37 compared to 9.412 95% CI 7.85 – 10.98, p < 0.0001) (**Figure 8**). A kaleidoscope score > 15 was associated with moderate/severe disease (OR 77 (95% CI 2.56 – 2312.22 p = 0.01) in the cohort of individuals with IEI.

## Discussion

The COVID-19 disease risk in the general population has improved with the development of highly effective vaccines. However, many individuals with immunodeficiency are still at risk of developing severe and refractory COVID-19 infection, potentially leading to the development of SARS-CoV-2 variants of concern (27). This study aimed to evaluate the vaccine response and COVID-19 disease severity of a group of 71 individuals with immunodeficiency. Our results show that whole-blood IGRA measuring IFN-γ levels post spike protein or peptide (S, S1, S+) mix stimulation is an accurate test to measure vaccine response to COVID-19 vaccination with no significant gender or age-related variability. Whole blood IGRA stimulation to peptide mixture is more sensitive in healthy controls and immunodeficient subjects than spike protein stimulation. This might be attributed to the process of degradation of whole spike protein and presentation of protein components *via* MHC molecules by the breadth of antigen-presenting cells (APCs) to mount an immune response. It is conceivable that the use of immunogenic peptides may bypass the requirement for processing prior to MHC presentation facilitating a different pathway to interaction with the T-cell receptor. Further work to establish the clinical relevance of this variation might be important in immunodeficiency settings and beyond. Whilst B-cells play a role as APCs, the exuberant response in XLA patients that lack peripheral blood B-cells upon stimulation with recombinant spike protein indicates that other APC cells are critical in this process. Most, but not all, individuals with IEI and SID had a cellular response and a good antibody level post-vaccine. Additionally, our study demonstrates that individuals with IEI have significantly improved IFN-γ response to peptide (S, S1,S+) and whole spike stimulation following the booster vaccine, indicating a benefit of the booster vaccine in terms of cellular immunity.

Notably, a higher IDDA 2.1 score and a vaccine response score of less than three were associated with worse COVID-19 disease severity in the small number of individuals that contracted the disease. The majority of individuals who developed mild COVID-19 showed evidence of a complete pre-infection vaccine response, whereby only one individual with moderate/severe COVID-19 showed evidence of cellular and humoral response to the vaccine. Four individuals with immunodeficiency and absent spike protein-specific antibody responses post vaccine developed mild symptoms only, suggesting an important role of the adaptive cellular immunity and the innate immune system in limiting SARS-CoV-2 viral replication. Interestingly, most patients with XLA in this cohort only developed mild disease. Nonetheless, small numbers of patients with XLA were included in this cohort, and one of these patients developed severe and refractory COVID-19 requiring intensive care treatment. In contrast, a disproportionate number of patients treated with B-cell-depleting therapies develop severe COVID-19 (28). This paradox may highlight the importance of monitoring immunoglobulin levels in individuals with hematologic malignancies or those receiving immunosuppressive therapies in addition to initiating long-term immunoglobulin replacement therapy where indicated in the setting of hypogammaglobulinaemia.

This study has several limitations. Firstly, it is important to note that the correlates of protection of IFN-γ levels to spike protein and peptide mix stimulation are unknown. Furthermore, even though it is likely that the majority of IFN-γ is T-cell derived, it is known that innate lymphoid cells produce IFN-γ to a lesser degree. It is also uncertain whether the source of IFN-γ is different between healthy controls and individuals with immunodeficiency. It is also important to note that the vaccine response score used in this study is an arbitrary unvalidated measurement, making it difficult to determine its utility outside of this study. This study is a single-centre real-world study with a relatively small, heterogenous sample size. Therefore, further studies will be needed to determine whether these results can be replicated in more focused cohorts with more detailed characterization of patients immunodeficiency.

Considering the nature of this study, other weaknesses include heterogeneity in sample timing and sample collection. Additionally, two different vaccine types were used for primary vaccination. Because many of the included individuals were maintained on immunoglobulin replacement therapy, it is important to consider that neutralizing antibodies in these products may affect these results. Some products tested from September 2021 had detectable anti-SARS-CoV-2 antibodies, therefore, it is not certain to which extent the antibodies detected in individuals with XLA and some individuals with CVID (**Figure 4**) originate from immunoglobulin replacement therapy. Lastly, it is possible that some of the included individuals may have contracted COVID-19 but did not inform the clinical team or did not have a symptomatic infection

Thus far, several studies have described the use of IGRA and immunoglobulin response to SARS-CoV-2 vaccination in various populations (1–15). Nevertheless, the protective value of a cellular response to the COVID-19 vaccine in the healthy population remains incompletely understood. Despite possible differences between cellular contributions between healthy individuals and individuals with immunodeficiency, whole-blood COVID-19 IGRA may offer a holistic way of quantifying a functional response to COVID peptides reflecting real and possibly significant functional deficits *in vivo*. The main strength of this study is that it examines in-depth the vaccine response in association with clinical features and COVID-19 disease in a cohort of individuals with immunodeficiency. Furthermore, this study offers robust validation of the utilized assays in young and elderly healthy controls. As far as we are aware, this is the first study investigating clinical immunodeficiency scoring and vaccine response scoring in individuals with immunodeficiency and COVID-19 disease.

To date, the utility of measuring the cellular immune response to vaccines in a healthy population has not been fully described. However, it is reasonable to assume that a positive antibody level alone may be falsely reassuring to vulnerable patients with cellular and combined immunodeficiencies. Therefore, we believe that the complete evaluation of cellular and humoral immunity gives a more accurate overview of individual disease risk and may be particularly informative for patients with immunodeficiency.

It is essential to develop harmonized methods for measuring cellular immunity to COVID-19 and determining their clinical utility. IGRA using whole blood is a straightforward, relatively cheap and reproducible assay already used in many diagnostic laboratories to measure IFN-γ response to tuberculosis and CMV (29). In addition, the ease of sample handling and validation of these assays make these methods easy adaptable to measure cellular response to COVID-19 (30).

In future studies, there may be a role for the IDDA 2.1 score, not only in describing the degree of immune dysregulation in IEI but also in estimating COVID-19 disease risk or the risk of other infectious diseases and identifying individuals who may benefit from enhanced vaccination schedules. A validated vaccine response score may be a valuable tool in assessing individuals’ level of protection from COVID-19 following vaccination and monitoring individuals’ COVID-19 vaccine response. Multi-institutional and collaborative studies between scientists and clinicians are needed to investigate vaccine responses and COVID-19 disease risk over the long term in individuals with immunodeficiency. These efforts will play a vital part in understanding risk factors and preventing severe COVID-19 in this population.

## Data availability statement

The original contributions presented in the study are included in the article/supplementary material. Further inquiries can be directed to the corresponding author.

## Ethics statement

The studies involving human participants were reviewed and approved by St. James’s Hospital Research Ethics Committee. Written informed consent for participation was not required for this study in accordance with the national legislation and the institutional requirements.

## Author contributions

Conceptualization, NC and JD. Methodology, NC, JD, CO ‘B, RA. Testing RK, MG, AN, EK, SN, PH, ASc. Sample collection and details of vaccinations provided, ASl, PM, JS and CM. Writing – original draft, CM, CO’B. Writing – review and editing, CM, CO’B, NC, JD, SA and SP. All authors contributed to the article and approved the submitted version.
